# Rigid-platform transanal excision (TEM/TEO/TAMIS) for rectal neuroendocrine tumours: a single-centre TEM/TEO series and systematic review

**DOI:** 10.1007/s13304-026-02621-x

**Published:** 2026-04-03

**Authors:** Alberto Arezzo, Carlo Alberto Ammirati, Giovanni Distefano, Michele Barbiero, Mario Morino

**Affiliations:** Department of Surgical Sciences, University of Turin, Città Della Salute E Della Scienza Hospital, Corso Bramante 88, 10126 Turin, Italy

**Keywords:** Rectal neuroendocrine tumours (rNETs), Transanal endoscopic microsurgery (TEM), Local excision, Salvage surgery, Neuroendocrine neoplasms (NENs)

## Abstract

Rectal neuroendocrine tumours (rNETs) are increasingly detected and often amenable to organ-preserving local therapy. Rigid transanal platforms (TEM/TEO/TAMIS) offer en bloc, full-thickness excision with precise margin control; however, contemporary evidence is limited. We report our single-centre outcomes and contextualise them with a focused, PRISMA-guided systematic review. We retrospectively analysed a prospectively maintained database of consecutive rNETs treated with TEM/TEO (1993–2025). The primary endpoint was R0 resection; secondary endpoints included complications, length of stay, residual tumour at completion excision, and oncologic outcomes. In parallel, we systematically searched PubMed/MEDLINE and Embase (last update August 1st 2025) for studies reporting outcomes of rigid transanal excision for rNETs (TEM/TEO/TAMIS/robotic TAMIS). Purely endoscopic series, radical resections, and case reports (< 5 patients) were excluded. Fourteen patients underwent local excision (median age 64 years; median distance from anal verge 7.5 cm). Tumour size was captured using two complementary metrics: preoperative endoscopic diameter (diameter pre) and histological diameter on the resected specimen (diameter histo), both reported in millimetres (mm). Median diameter pre was 20 mm (IQR 20–30; range 20–100), while median diameter histo was 30 mm (IQR 20–30; range 10–60). Histology revealed 10 G1 and 4 G2 tumours; lymphovascular invasion was present in 2 (14.3%). All procedures were completed transanally; the median operative time was 52 min, and the median stay was 2 days. One minor complication (Clavien–Dindo II) occurred. R0 resection was achieved in 14/14 (100%); among five completion excisions, residual tumour was present in 2 (40%). Over a median follow-up of 36 months (IQR 26–48; range 12–72), one patient (7.1%) recurred (nodal) and underwent salvage total mesorectal excision. The systematic review included 11 studies (~ 440 unique patients). Across series, R0 rates were typically 96–100%, morbidity was low (< 10%, mostly minor), and residual tumour at completion occurred in ~ 20–40% after incomplete endoscopic resection. Recurrences were uncommon but could occur late (> 10 years). Rigid transanal excision achieves high-quality local control with low morbidity and provides reliable full-thickness clearance after incomplete endoscopic resection. The combined presentation of our institutional series with a focused review clarifies where TEM/TEO/TAMIS most benefits rNET care—particularly for completion surgery and intermediate-risk lesions—within contemporary guideline frameworks.

**Supplementary Information:**

The online version contains supplementary material available at 10.1007/s13304-026-02621-x.

## Introduction

Rectal neuroendocrine tumours (rNETs) are increasingly recognised in daily clinical practice, mainly as a consequence of the widespread adoption of screening colonoscopy and improved histopathological classification. Rather than being rare incidental findings, rNETs are now among the most frequently diagnosed gastrointestinal neuroendocrine neoplasms in contemporary registries, with rising detection largely driven by screening and improved pathology reporting [1, 2]. This increase is attributable not only to screening but also to enhanced awareness and diagnostic accuracy, leading to the detection of small, incidentally discovered lesions that were previously overlooked.

Despite their relatively indolent natural history, rNETs display heterogeneous biological behaviour. Prognosis is strongly conditioned by tumour size, grade, depth of invasion, and the presence of lymphovascular invasion (LVI). According to the current WHO classification, the majority are well-differentiated grade 1 or 2 tumours, whereas grade 3 neoplasms and poorly differentiated carcinomas are distinctly uncommon [3]. Risk of nodal involvement is minimal in tumours ≤ 10 mm, rises to approximately 10–15% in the 10–20 mm range, and exceeds 50% in tumours larger than 20 mm [4–6]. In addition to size, adverse histological features such as LVI, higher grade, and infiltration of the muscularis propria confer a significant increase in metastatic potential [7].

Management of rNETs is therefore stratified by these prognostic determinants. The most recent ENETS guidance recommends endoscopic excision for lesions ≤ 10 mm confined to the submucosa, individualised decision-making for those between 10 and 20 mm depending on grade, LVI, and depth of invasion, and formal oncologic resection for lesions > 20 mm or in the presence of nodal disease [8]. The NCCN guidelines provide comparable recommendations, emphasising the role of multidisciplinary evaluation in borderline cases [9]. The therapeutic dilemma is most pronounced for intermediate lesions measuring 10–20 mm, where both local and radical strategies may be justifiable, and where institutional expertise often influences treatment decisions [10].

Endoscopic techniques have evolved considerably in response to this challenge. While conventional EMR is limited by high rates of incomplete resection in tumours > 5 mm, modifications such as cap-assisted or band ligation EMR, as well as ESD, have substantially improved en-bloc and R0 rates, particularly in expert centres [11–14]. Endoscopic full-thickness resection (eFTR) using over-the-scope clip devices has also emerged as a potential alternative, with promising early outcomes [15]. Yet, even in high-volume series, incomplete resection and positive margins remain clinically relevant. Several surgical case series confirm that histologic residual tumour is found in 20–40% of patients undergoing transanal re-excision after an incomplete endoscopic attempt [16–18]. This highlights the persistent need for a complementary, surgically based strategy.

Rigid-platform transanal surgery provides such an option. Since its introduction by Buess in the 1980s [19], transanal endoscopic microsurgery (TEM) has offered unparalleled visualisation, stable pneumorectum, and dedicated instrumentation to perform precise, full-thickness excision throughout the rectum. Its successor, the transanal endoscopic operation (TEO), and the later development of transanal minimally invasive surgery (TAMIS) [20] have further broadened access to local excision, providing flexible, cost-effective alternatives while maintaining the principle of en bloc, full-thickness resection. Across multiple retrospective series, TEM and TAMIS have consistently yielded R0 rates exceeding 95%, minimal morbidity, and rare recurrences, with occasional reports of late failures beyond a decade [21–25]. Vital is their role in completing excision after endoscopic resection, as up to one-third of such cases harbour residual tumour, underscoring the oncological security conferred by rigid transanal platforms [16, 18, 24]. Robotic TAMIS has recently been explored, with encouraging short-term outcomes; however, evidence specific to NET remains limited [26].

Given the long study period, we also summarised key practice changes by era (1993–2007 vs 2008–2025), including the transition from TEM to TEO and the progressive adoption of modern staging (EUS and pelvic MRI) and contemporary WHO grading criteria (Supplementary Table S1).

Despite these favourable data, evidence remains fragmented, with heterogeneous inclusion criteria, variable follow-up, and relatively small sample sizes in most reports. Comparative data between rigid-platform excision and advanced endoscopic methods are sparse, and long-term oncologic outcomes are inconsistently documented. As a result, the contemporary role of TEM/TEO/TAMIS in rNETs—particularly in Western practice where ESD expertise is less prevalent—remains to be clarified. Practice variation in transanal management strategies has also been documented in recent surveys, underscoring the need for transparent reporting and pragmatic selection frameworks [10].

The aim of the present study is therefore twofold. First, we provide a comprehensive synthesis of the available literature on rigid-platform transanal excision (TEM, TEO, TAMIS) for rectal neoplasms (rNETs), conducted in accordance with the PRISMA methodology. Second, we report our institutional series of 14 patients treated with TEM/TEO over more than two decades, analysing perioperative outcomes, completeness of resection, residual disease in completion procedures, complications, recurrence, and long-term oncological control. By combining institutional experience with a focused review and contrasting rigid-platform surgery with advanced endoscopic techniques in light of the ENETS 2023 recommendations, we aim to define the present and future role of TEM/TEO/TAMIS in the management of rectal NETs more clearly.

## Materials and methods

### Systematic review

**Fig. 1:**
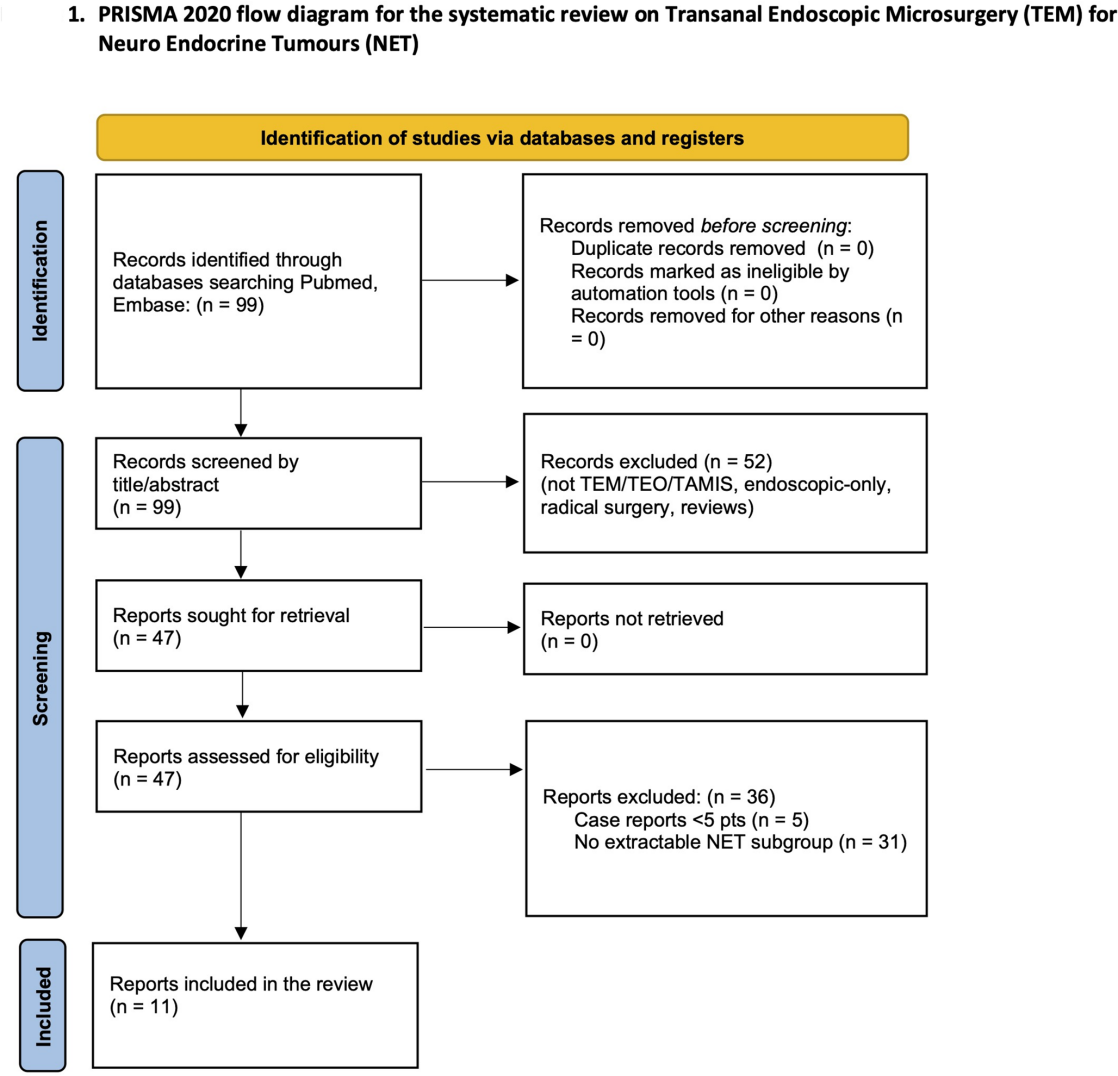
PRISMA 2020 flow diagram illustrating the study selection process for the systematic review on Transanal Endoscopic Microsurgery (TEM) for rectal neuroendocrine tumours (NETs)

A systematic review was conducted in accordance with the PRISMA 2020 guidelines (Fig. 1).

A comprehensive search of PubMed/MEDLINE and Embase was performed (last updated August 1st 2025). The PubMed search combined free-text and controlled vocabulary (MeSH) for rectal NET/carcinoid and rigid transanal platforms. The free-text component was:

("rectal neuroendocrine tumour" OR "rectal neuroendocrine tumor" OR "rectal neuroendocrine neoplasm" OR “rectal carcinoid”) AND ("transanal endoscopic microsurgery" OR TEM OR "transanal endoscopic operation" OR TEO OR "transanal minimally invasive surgery" OR TAMIS OR "transanal endoscopic surgery" OR TES OR (robotic AND TAMIS)). For Embase, the following Emtree/free-text strategy (title/abstract/keyword) was used: ('rectum neuroendocrine tumor’/exp OR 'rectal neuroendocrine tumor’:ti,ab,kw OR 'rectal neuroendocrine tumour’:ti,ab,kw OR 'rectal neuroendocrine neoplasm’:ti,ab,kw OR ‘rectal carcinoid’:ti,ab,kw) AND ('transanal endoscopic microsurgery’/exp OR 'transanal endoscopic operation’:ti,ab,kw OR TEM:ti,ab,kw OR TEO:ti,ab,kw OR 'transanal minimally invasive surgery’:ti,ab,kw OR TAMIS:ti,ab,kw OR 'transanal endoscopic surgery’:ti,ab,kw OR TES:ti,ab,kw OR 'robotic transanal minimally invasive surgery’:ti,ab,kw).

No language or year restrictions were applied; bibliographies of relevant papers were screened manually.

**Fig. 2:**
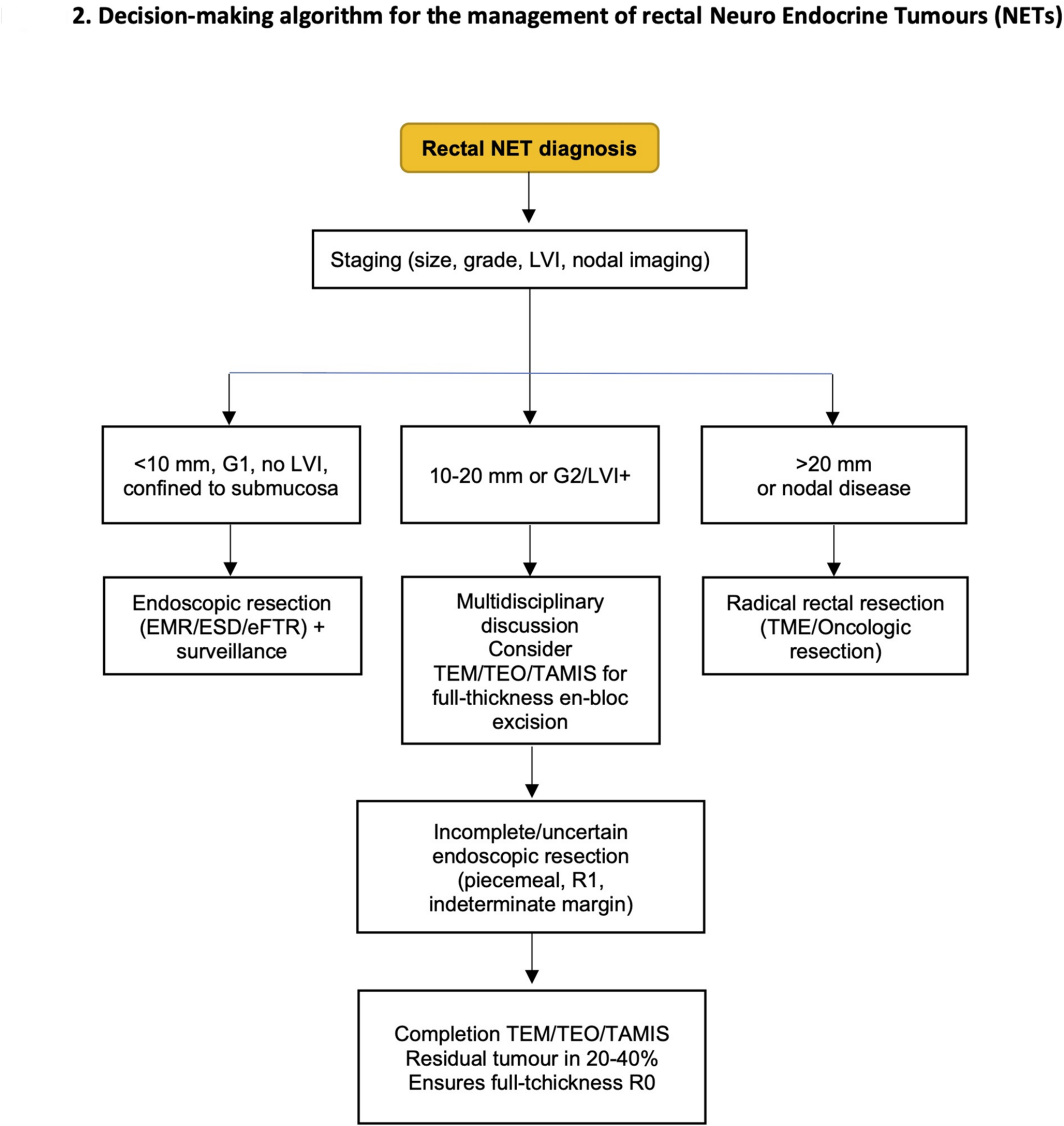
Clinical decision-making algorithm for the management of rectal neuroendocrine tumours (NETs)

Included were studies reporting outcomes of rigid-platform transanal approaches (TEM, TEO, TAMIS, robotic TAMIS) for rectal neuroendocrine tumours (rNETs), either as primary treatment or as completion after incomplete endoscopic excision. Excluded were case reports (< 5 patients), purely endoscopic resections (EMR, ESD, eFTR), radical rectal resections, and reviews without original data (Fig. 2).

Two reviewers (CAA, GD) independently screened abstracts and full texts. Data were extracted into a structured form including: author, year, journal, country/setting, number of patients, indications (primary vs completion), tumour size, distance from anal verge, operative details, complications, margin status (R0), residual tumour at re-excision, follow-up duration, and recurrence/metastatic outcomes. Additional fields included tumour grade (according to the WHO classification) and the presence of lymphovascular invasion (LVI), when reported. Disagreements were resolved by discussion; if needed, a third author adjudicated.

Potential overlap between cohorts was assessed by centre, recruitment period, and author lists; when overlap was suspected, the most comprehensive or most recent dataset with the longest follow-up was retained to avoid double-counting.

Cohort and comparative studies were assessed using the ROBINS-I; case series were evaluated using the JBI checklist for case series. Results are presented descriptively given heterogeneity.

Due to heterogeneity, results were synthesised narratively with descriptive summaries of R0 resection, complications, residual tumour in completion excision, and oncologic events.

### Institutional case series

#### Study design and setting

We performed a retrospective review of our prospective TEM/TEO database maintained at the Department of General Surgery, University of Turin, covering the period 1993 to 2025. This database includes all consecutive patients undergoing rigid-platform transanal endoscopic surgery (TEM, TEO) for rectal lesions. For this analysis, only patients with a final histopathological diagnosis of rectal neuroendocrine tumour (NET) were included.

This study was retrospective and based on routinely collected clinical data. All data were fully anonymised prior to analysis. According to applicable national regulations and institutional policy for retrospective studies using anonymised data, formal Ethics Committee approval and individual informed consent were not required.

#### Patient selection

Patients were eligible if they underwent primary local excision for a suspected NET or completion excision after an incomplete endoscopic removal (R1 or piecemeal EMR/ESD). Exclusion criteria were: (i) histology other than NET; (ii) NETs treated by radical rectal resection. For oncologic endpoints, we required a minimum follow-up of 6 months to allow at least one post-treatment assessment; perioperative outcomes were recorded for all eligible patients.

#### Preoperative work-up

All patients underwent digital rectal examination and endoscopy. Tumour size and location (distance from anal verge) were recorded. Specifically, lesion diameter was recorded as a preoperative endoscopic estimate (diameter pre) and as the maximum diameter on the resected specimen (diameter histo); both are reported in millimetres (mm) after conversion from the original centimetre-based dataset (× 10). Staging evolved over decades; in recent years, this included endorectal ultrasound and pelvic MRI for lesions ≥ 10 mm or suspicious for muscularis propria invasion, in line with ENETS guidelines.

#### Surgical technique

Procedures were performed with Buess’ TEM platform (Richard Wolf) until 2008 and with TEO (Karl Storz) thereafter. Both systems provide rigid access with CO₂ pneumorectum, magnified vision, and allow full-thickness surgical excision. Excision intent was a full-thickness, en bloc resection with a margin of healthy tissue surrounding the lesion. Defects were closed with running long-lasting absorbable sutures when feasible. Type of anaesthesia (general vs spinal), operative time, and intraoperative events (e.g., peritoneal opening, conversion) were recorded.

#### Histopathological evaluation

Specimens were oriented, pinned, and assessed by dedicated gastrointestinal pathologists. Tumour size, grade (WHO classification), depth of invasion, presence of lymphovascular invasion (LVI), and margin status were recorded. R0 resection was defined as negative lateral and deep margins.

#### Postoperative management and follow-up

Patients were monitored for complications during hospital stay. Complications were graded according to Clavien-Dindo classification.

Follow-up consisted of clinical examination, endoscopy, and imaging (pelvic MRI and/or endorectal ultrasound), scheduled according to ENETS guidelines and tailored to tumour size, grade, and LVI status. Local recurrence, nodal and distant metastases, and survival outcomes were documented.

#### Endpoints

The primary endpoint was the rate of R0 resection. Secondary endpoints included operative time, complications, hospital stay, recurrence/metastasis, and pathological risk factors (tumour size, grade, and lymphovascular invasion).

## Results

### Institutional case series

Between 1993 and 2025, 14 patients with rectal neuroendocrine tumours (rNETs) underwent local excision using rigid transanal platforms (TEM, TEO) at our institution. Table 1 summarises the findings of the Institutional Case Series.

**Table 1:** Patient characteristics–Institutional Case Series (n = 14). Tumour size is reported as preoperative endoscopic diameter (diameter pre) and histological diameter on the resected specimen (diameter histo), in mm (converted from the original cm-based dataset × 10)

Case	Age	Sex	Tumor location (cm from AV)	Diameter pre (mm)	Diameter histo (mm)	Grade (WHO)	LVI	Margin status	Complications	Follow-up (months)	Recurrence	Reoperation
1	53	M	6	20	10	G1	neg	R0	No	24	No	No
2	70	M	5	20	10	G1	neg	R0	No	36	No	No
3	64	F	12	20	30	G1	neg	R0	No	18	No	No
4	61	F	4	50	20	G2	pos	R0	No	48	No	No
5	46	M	8	100	20	G1	neg	R0	No	60	No	No
6	67	F	9	20	30	G2	neg	R0	No	30	No	No
7	58	F	7	20	30	G1	neg	R0	No	40	No	No
8	76	M	8	20	30	G1	neg	R0	No	12	No	No
9	65	F	3	30	30	G2	pos	R0	Yes (Clavien II)	36	Yes (LN)	Yes (TME)
10	63	M	6	20	20	G1	neg	R0	No	28	No	No
11	68	M	12	20	20	G1	neg	R0	No	72	No	No
12	64	M	10	20	30	G1	neg	R0	No	54	No	No
13	48	F	9	30	60	G2	neg	R0	No	20	No	No
14	77	F	3	70	50	G1	neg	R0	No	48	No	No

AV, anal verge; LN, lymph node; LVI, lymphovascular invasion; NR, not reported; R0, complete resection (margine negative); TEM, transanal endoscopic microsurgery; WHO, World Health Organization

#### Demographics and tumour characteristics

The median patient age was 64 years (IQR 59.5–67.5; range, 46–77), with 7 males and 7 females. Tumours were located at a median distance of 7.5 cm from the anal verge (IQR 5.5–9.0; range 3–12). Tumour size is reported as both diameter pre and diameter histo (in mm). Median diameter pre was 20 mm (IQR 20–30; range 20–100), while median diameter histo was 30 mm (IQR 20–30; range 10–60). According to the WHO classification, 10 tumours were classified as G1 (71.4%) and 4 as G2 (28.6%). Lymphovascular invasion (LVI) was present in 2 patients (14.3%).

Depth of invasion was confined to the submucosa in all cases; no tumours showed muscularis propria invasion on final pathology.

#### Operative details

All procedures were completed transanally without the need for conversion. Spinal anaesthesia was used in 9 cases (64.3%) and general anaesthesia in 5 (35.7%). The median operative time was 52 min (range, 37–80 min). Peritoneal entry occurred in 2 patients (14.3%); in both cases, the defect was closed primarily with running sutures, with no further consequences.

#### Postoperative outcomes

The median hospital stay was 2 days (range, 1–7); 4 patients (28.6%) were discharged within 24 h of admission. One patient experienced minor postoperative morbidity (Clavien–Dindo II). No readmissions or reoperations for complications were necessary.

#### Pathology

Overall, R0 resection was achieved in all patients (14/14, 100%). Among the five completion excisions after prior incomplete endoscopic resection, residual tumour was found in 2 cases (40%).

#### Follow-up and oncological outcomes

During a median follow-up of 36 months (IQR 26–48; range, 12–72 months), one patient (7.1%) developed nodal recurrence and underwent salvage total mesorectal excision. No disease-related deaths were recorded during follow-up.

### Systematic review

From an initial yield of 99 records, 47 full-text articles were assessed, and 11 met the predefined inclusion criteria [16–18, 21–26, 28, 29]. These studies, published between 2005 and 2023, reported on approximately 440 patients with rectal neuroendocrine tumours (rNETs) undergoing excision with rigid-platform transanal techniques, including TEM, TEO, TAMIS, robotic TAMIS, or their precursor, TES. Most were single-centre, retrospective series; two were multicenter, and one provided a direct comparison with endoscopic submucosal dissection (ESD). Table 2 summarises findings of the Systematic Review.

**Table 2:** TEM/TEO/TAMIS studies and reports on rectal NET excision

Author	Year	Journal	n	Technique and setting	Diameter (mm)	Distance from AV (cm)	Operative time (min)	Hospital stay (days)	Morbidity	R0 (%)	Residual tumour (completion)	Follow-up (months)	Oncologic events	Grading (WHO)	LVI	Indication criteria
Ishikawa	2005	Surg Endosc	17	TES; primary	8.8 ± 2.9	6.8 (mean)	76.6 ± 27.3	5.2 ± 2.8	NR	100	–	74.2 ± 47.5	0 recurrence	G1–2	NR	NET ≤ 2 cm, G1–2
Kinoshita	2007	Surg Endosc	27	TEM; 14 primary, 13 completion	9.1 ± 4.1	8.5 ± 3.7	51.6 ± 16.6	—	7.4%	100	30.8%	70.6 ± 43.8	0 recurrence	G1–2	NR	NET ≤ 2 cm, no LVI
Kumar	2011	Colorectal Dis	24	TEM; primary + completion	9.3 (mean)	7.5 (mean)	52 (mean)	1.6 (mean)	0	96 (23/24)	17%	72 (med.)	1 local recurrence (54m)	G1 prevalent	3 cases LVI +	NET ≤ 2 cm, G1–2
Kim	2012	JKSC	38	TEM; primary + completion	NR	NR	NR	NR	0 severe	97.4	–	75 ± 33	1 liver metastasis; 1 LN recurrence	G1–2	some LVI +	NET ≤ 2 cm, G1–2
Chen	2015	WJG	59	TEM full-thickness; 38 primary, 21 post-endo	7.2 ± 3.0	8.4 ± 2.0	57.6 ± 19.0	2.7	minor (bleeding)	100	0%	≈36	0 recurrences	G1–2	10% LVI +	NET ≤ 2 cm, G1–2
Tomassi	2019	Dis Colon Rectum	58	robot-TAMIS; mixed series	NR	8.8 (mean)	66.2 (mean)	89.7% DS	5.2% minor	94.8 overall	–	11.5 (mean)	recurrences overall (5.5%)	NR	NR	Various T1–2 (not only NET)
Kang	2020	JMIS	18	TAMIS; subgroup NET	16 (mean)	7.0 (mean)	52.1 ± 33.5	4.3 ± 4.2	13.3% minor	96.7 overall	–	NR	NR	G1–2	NR	NET ≤ 2 cm, G1–2
Hayashi	2021	Surg Endosc	10	TAMIS; 6 primary, 4 completion	8 (mean); range 5–15	5.3 (mean)	80.5 (mean)	7 (mean)	0 major	100	0%	54 (med.)	0 recurrences	G1	0	NET ≤ 2 cm, G1
Park	2021	GIE	45	TAMIS vs ESD; ≤ 20 mm	11.6 (mean)	NR	NR	NR	NR	95.6	–	33 (med.)	1 recurrence (2.2%)	G1–2	some LVI +	≤ 2 cm, G1–2
Shi	2022	BMC Surg	144	TEM; multicentric	6 (mean); range 3–20	NR	NR	NR	2.1%	98.6 (142/144)	–	75.5 (med.)	2 metastasis	G1–2	8% LVI +	NET ≤ 2 cm
Lie	2023	Colorectal Dis	58	TEM; 15 primary, 38 completion, 5 re-recurrence	7.4 ± 3.8	6.6 (mean)	37.2 ± 17.2	96.6% DS	6.9% minor	100	21.1%	Up to 150	3 recurrences (2.1–12.5 year)	G1–2	10% LVI +	≤ 2 cm, G1–2

AV, anal verge; ESD, endoscopic submucosal dissection; LN, lymph node; LVI, lymphovascular invasion; med., median; n, number of cases; NET, neuroendocrine tumour; NR, not reported; R0, complete surgical resection; TAMIS, transanal minimally invasive surgery; TEM, transanal endoscopic microsurgery; TEO, transanal endoscopic operation; TES, transanal endoscopic surgery; WHO, World Health Organization

Risk-of-bias assessment suggested an overall moderate risk of bias across most retrospective cohorts (mainly due to confounding and outcome measurement), while reporting quality in case series was generally acceptable but variable; detailed domain judgements are provided in Supplementary Table S2.

*Patient and tumour characteristics* were consistent across studies. Median patient age ranged between the mid-fifties and mid-sixties, with a balanced sex distribution. Median tumour size was between 6 and 12 mm, with the majority located 4–10 cm from the anal verge. The overwhelming majority of tumours were WHO grade 1, although 10–25% of patients harboured grade 2 tumours. Lymphovascular invasion (LVI) was identified in 8–15% of cases when systematically reported. Indications for surgery were either primary local excision of a suspected rNET or completion excision after an incomplete endoscopic resection; the proportion of completion resections varied widely across series, ranging from 25 to 75% in older cohorts and remaining relevant in more recent ones.

*Operative outcomes* were favourable throughout. Median operative time ranged from 37 to 80 min, with spinal anaesthesia commonly used in TEM/TEO and general anaesthesia more often used for TAMIS. Hospital stay was progressively reduced over time: while earlier studies reported median stays of 3–5 days, more recent series achieved day-case discharge or ≤ 48-h admission in over half of patients. Intraoperative peritoneal entry was occasionally encountered, affecting approximately 2–5% of cases, but could be managed safely with primary closure, without need for conversion or adverse sequelae. Postoperative morbidity was consistently low, generally below 10% across series and nearly always minor (urinary retention, self-limiting bleeding, transient anal pain). No mortality was reported.

*Pathological outcomes* confirmed the strength of rigid-platform transanal excision. Across nearly all studies, R0 resection rates exceeded 95%, frequently reaching 100%. In the most extensive series (Shi et al. n = 144), 98.6% of resections were margin-negative. Similar rates were reported in smaller but dedicated cohorts from Japan, China, and Canada. A recurring finding was the detection of residual tumour in completion specimens after endoscopic R1 or piecemeal resection, ranging from 20 to 40% in several series. This highlights the oncological importance of rigid-platform re-excision in cases of incomplete endoscopic removal.

*Oncological outcomes* were excellent overall. Median follow-up across studies ranged from 33 to 75 months, with several extending beyond 5 years and one study (Lie 2023) documenting recurrences after more than 12 years. Recurrence rates were generally low, ranging from 2 to 5%. Most failures were local, although distant metastases were occasionally observed in G2 or LVI-positive tumours (e.g., Kim 2012, Shi 2022). Radical salvage surgery, usually by total mesorectal excision, was performed in these cases and achieved secondary cure. Importantly, late failures were documented, underlining the need for long-term follow-up despite excellent initial results. No disease-related deaths were reported in any of the included series.

Robotic TAMIS was explored in two reports. Tomassi [26] reported a mixed rectal neoplasia cohort (NET-specific outcomes not isolated) demonstrating feasibility, high rates of margin negativity, low morbidity, and frequent same-day discharge. NET-specific robotic data remain sparse and were interpreted as feasibility evidence rather than comparative oncologic outcomes.

## Discussion

Our experience adds to a decades-long arc showing that rigid transanal platforms can deliver reliable, organ-preserving treatment for rectal NETs. Since the pioneering work that established a stable pneumorectum and magnified vision for full-thickness local excision [19], and the subsequent diffusion of TAMIS as a more flexible access strategy [20], multiple series have reported high rates of complete excision with low morbidity. In this context, our cohort mirrors the prevailing signal: an overwhelmingly curative local procedure, a short and uncomplicated postoperative course, and durable control, with the caveat—shared by others—that late failures can occur and warrant long-term surveillance.

Placed alongside contemporary evidence, our outcomes are directionally consistent. Historical and modern TEM/TAMIS series typically report R0 rates ≥ 95% and very low local failure, including the Japanese and North American cohorts by Kinoshita and Kumar [16, 17], the considerable single-center Chinese experience by Chen [21], and the recent Canadian series by Lie, which achieved 100% R0 yet still documented three recurrences—one beyond a decade [18]. We observed similarly high rates of radicality, minor morbidity, and infrequent recurrence, which reinforces that rigid transanal excision remains an oncologically sound approach when used in the appropriate window.

A recurrent theme across the literature—and echoed in our practice—is what rigid platforms reveal after incomplete endoscopic resection. Several studies demonstrate that completion TEM/TAMIS identifies histologic residual tumour in approximately one-fifth to one-third of cases [16–18, 21]. Our completion specimens behaved no differently. This matters clinically: an apparently “clean” scar can harbour residual microfoci, and only a full-thickness, en-bloc specimen settles the deep margin and permits confident staging, including assessment of lymphovascular invasion (LVI) and grade—variables repeatedly implicated in risk stratification across epidemiological and guideline frameworks [1–3, 7–9].

Technically, the advantage of TEM/TEO is straightforward to articulate to a multidisciplinary team: stable exposure; controlled, perpendicular deep margin; specimen orientation; and primary closure when the peritoneum is entered—all with low morbidity and rapid recovery [27]. Modern TAMIS reproduces many of these virtues through a softer portal, and the focused rNET series now documents excellent performance—Hayashi reported 100% R0 with no recurrences at a median 54 months [23]. Robotic TAMIS enhances ergonomics and suturing comfort, although NET-specific data remain limited and primarily focused on feasibility [26]. Broader endoluminal robotic platforms are also rapidly evolving and have been appraised using the IDEAL framework, highlighting early-stage evidence and the need for structured evaluation and longer-term outcomes [27].

At the same time, endoscopic therapy has advanced dramatically, particularly in Asia. Modified EMR techniques and ESD can achieve en bloc resection with excellent outcomes for very small (≤ 10 mm) G1 rNETs when delivered by experienced teams [14]. For these tiny, favourable lesions in expert hands, outcomes approach those of rigid transanal surgery while avoiding a full-thickness defect. The distinction becomes sharper in the grey zone: 10–20 mm, G2 histology, suspected submucosal extension, or any scenario in which en bloc deep margin control is uncertain. In those patients—more common in Western programs with variable ESD penetration—rigid transanal excision provides a reproducible oncologic solution, with consistently high R0 and intact specimens for definitive pathology [16–18, 21, 23, 24]. Recent comparative analyses have further compared modern endoscopic techniques for rectal NETs, supporting technique selection for small, low-risk lesions [28].

Current guidance synthesises this nuance. ENETS (2023) and NCCN (2024) converge on a pragmatic size- and risk-adapted approach: endoscopic resection for ≤ 10 mm well-differentiated tumors if actual en-bloc R0 is achievable; oncologic resection for ≥ 20 mm or nodal disease; and individualized decision-making for the 10–20 mm interval, where platform choice should hinge on depth, grade, LVI, and local expertise [8, 9]. Our series sits squarely within this framework. We used rigid platforms both upfront and for completion when endoscopy was piecemeal or R1; we achieved high R0 rates; and we observed excellent disease control, acknowledging that late events—also seen by Lie—can surface and justify long-term follow-up [18].

The study’s limitations are inherent to the disease and its design, including a small sample size, single-centre setting, and retrospective analysis. Over three decades, staging and pathology evolved (EUS/MRI adoption, WHO updates), introducing heterogeneity [3, 8, 9]. We did not capture formal functional outcomes (continence, quality of life), and we did not conduct a head-to-head comparison with ESD at our institution. Still, strengths include a prospectively maintained transanal database, uniform full-thickness intent, detailed pathology (including grade and lymphovascular invasion, or LVI), and extended follow-up—elements that align our observations with the most informative series in the field [16–18, 21, 23, 24].

Clinically, we propose a pragmatic, hybrid strategy that integrates endoscopic and transanal platforms. Given the heterogeneity of the evidence base, this approach should be individualised and validated in larger cohorts. A possible flow is as follows:

i.  ≤ 10 mm, G1, no LVI, favorable location suitable for high-quality endoscopic en-bloc R0 with structured surveillance [11–13, 15, 29];
ii. Incomplete/uncertain endoscopic resection (piecemeal, R1, indeterminate deep margin) should undergo completion TEM/TEO/TAMIS to secure full-thickness clearance and definitive staging [16–18, 21, 23];
iii.  ≥ 10 mm and/or G2/LVI + or suspected deep invasion requires multidisciplinary discussion; either en-bloc endoscopic resection in expert centres or rigid transanal excision can be considered depending on depth assessment and local expertise [8, 9, 18, 23, 24];
iv. nodal disease is indicated for oncologic rectal resection per guidelines [8, 9].

This shared-care model reflects where the field has arrived: endoscopy leads for tiny, favourable tumours; rigid transanal surgery anchors the intermediate-risk band and rescues uncertain endoscopic results; radical surgery is reserved for clearly advanced biology.

## Conclusions

Rigid-platform transanal excision delivers high rates of R0 resection with low morbidity and durable local control, with late failures rare but documented [16–18, 21, 23, 24]. Within ENETS and NCCN guidance, TEM/TEO (and TAMIS) remains particularly useful when endoscopic excision is incomplete or oncologically uncertain, and in selected 10–20 mm or higher-risk lesions where full-thickness, en-bloc margin control and definitive pathology are decisive [8, 9, 18, 21, 23, 24]. In the future, multicenter prospective registries and harmonised outcome reporting (grade, LVI, depth, functional metrics) are needed to refine selection and long-term surveillance while preserving rectal function in this growing patient population.

## Supplementary Information

Below is the link to the electronic supplementary material.

Supplementary Material 1

Supplementary Material 2

## Data Availability

All data generated or analysed during this study are included in this published article.
