# Supplementary material for: Rigid-platform transanal excision (TEM/TEO/TAMIS) for rectal neuroendocrine tumours: a single-centre TEM/TEO series and systematic review
Source: Updates Surg. 2026 Apr 3;78(3):1117–27. doi: 10.1007/s13304-026-02621-x (PMC13249663; doi:10.1007/s13304-026-02621-x)
Supplement: Supplementary file 1 — Supplementary Material 1 [file 13304_2026_2621_MOESM1_ESM.docx]

**Supplementary Table S1.** Era-stratified institutional practice (1993–2025)

*This table summarises, at a descriptive level, how diagnostic workup, platform availability and reporting standards evolved over the study period at our institution. Where historical details were not consistently recorded, items are reported as 'not routinely available/recorded'.*

| Era | Preoperative staging (typical/standard practice) | Platform/technique | Pathology reporting (typical) | Endoscopic alternatives (availability) |
| --- | --- | --- | --- | --- |
| 1993–2007 | Clinical assessment and rigid proctoscopy; colonoscopy. Cross-sectional imaging and endorectal ultrasound (EUS) used selectively when available; pelvic MRI not routinely performed in early years. | TEM introduced and used for selected rNETs and completion resections after endoscopic attempt. | WHO grading terminology evolved over time; Ki-67 index not routinely reported in early years. Margin status reported; depth of invasion variably described in historical reports. | Conventional EMR/ESD not widely available; endoscopic full-thickness resection (EFTR) not available. |
| 2008–2025 | Systematic staging with high-resolution pelvic MRI and/or EUS for lesions approaching or exceeding 10–20 mm, suspected deep invasion, or high-risk features; multidisciplinary discussion increasingly used for borderline cases. | Transition from TEM to TEO (platform upgrade); full-thickness excision routinely aimed when transanal excision indicated. | Contemporary WHO grading (G1/G2) and Ki-67-based assessment routinely reported; depth of invasion and LVI more consistently documented; margin status reported with emphasis on deep margin in full-thickness specimens. | ESD/EFTR and advanced endoscopic techniques became available, informing a platform-integrated treatment strategy (endoscopy vs transanal excision) based on risk profile and local expertise. |
