# Supplementary material for: Rigid-platform transanal excision (TEM/TEO/TAMIS) for rectal neuroendocrine tumours: a single-centre TEM/TEO series and systematic review
Source: Updates Surg. 2026 Apr 3;78(3):1117–27. doi: 10.1007/s13304-026-02621-x (PMC13249663; doi:10.1007/s13304-026-02621-x)
Supplement: Supplementary file 2 — Supplementary Material 2 [file 13304_2026_2621_MOESM2_ESM.docx]

**Supplementary Table S2.** Risk-of-bias assessment of included studies (systematic review)

*Risk of bias was assessed using a structured checklist for case series/observational studies (e.g., JBI case series domains). Judgements are based on reporting within each publication; 'Unclear' indicates insufficient reporting rather than absence.*

| Study | Selection/ inclusion criteria | Consecutive inclusion | Outcome ascertainment | Follow-up adequacy | Confounding control/ comparators | Overall judgement |
| --- | --- | --- | --- | --- | --- | --- |
| Ishikawa 2005 | Clear | Unclear | Adequate | Adequate | Not applicable | Moderate |
| Kinoshita 2007 | Clear | Unclear | Adequate | Adequate | Not applicable | Moderate |
| Kumar 2011 | Clear | Unclear | Adequate | Adequate | Not applicable | Moderate |
| Kim 2012 | Unclear | Unclear | Adequate | Adequate | Not applicable | Moderate |
| Chen 2015 | Clear | Unclear | Adequate | Moderate | Not applicable | Moderate |
| Tomassi 2019 | Unclear | Unclear | Unclear (NET subgroup) | Short/unclear | High (mixed indications) | High |
| Kang 2020 | Unclear | Unclear | Unclear (NET subgroup) | Unclear | High (subgroup) | High |
| Hayashi 2021 | Clear | Unclear | Adequate | Adequate | Not applicable | Moderate |
| Park 2021 | Clear | Unclear | Adequate | Adequate | Some (comparative design) | Moderate |
| Shi 2022 | Clear | Unclear | Adequate | Adequate | Limited | Moderate |
| Lie 2023 | Clear | Unclear | Adequate | Adequate | Not applicable | Moderate |
